# Cross-cultural validation of the self-assessment of competence in communication – SACCom – to Italian

**DOI:** 10.1590/2317-1782/e20250281en

**Published:** 2026-08-03

**Authors:** Aurora d’Apolito, Vanessa Veis Ribeiro, Glaucya Madazio, Mara Behlau

**Affiliations:** 1 Centro de Estudos da Voz – CEV - São Paulo (SP), Brasil.

**Keywords:** Clinical protocols, Self-testing, Speech, Language and Hearing Sciences, Communication, Voice, Speech, Interpersonal Relations

## Abstract

**Purpose:**

To carry out the cross-cultural validation of the Self-Assessment of Competence in Communication – SACCom to Italian.

**Methods:**

The cross-cultural validation procedure followed the recommendations and guidelines of the COnsensus-based Standards for the selection of health Measurement INstruments (COSMIN) and consisted of five stages: 1) Translation of the SACCom from Brazilian Portuguese (BP) into Italian by three bilingual speech-language-hearing pathologists; 2) Preparation of a consensus version; 3) Back-translation from Italian into BP by a fourth bilingual speech-language-hearing pathologist; 4) Comparison of the back-translation with the original instrument and preparation of the final version in Italian called *Test di Autovalutazione della Competenza nella Comunicazione* (TACCom-IT); and, 5) Application of the TACCom-IT pre-test to 101 native Italians whose voice and communication are essential to their profession. At this stage, the option “not applicable” was added to the answer key.

**Results:**

In the translation, there were different lexical choices regarding the title, instructions, and seven items. In the back-translation, there were conceptual disagreements in the title and instructions, and lexical disagreements in five items. The expert committee made changes to the title, instructions, and three items. In the pre-test phase, a significantly higher proportion of participants responded with options from the instrument's usual answer key for all items.

**Conclusion:**

The Italian adapted version of SACCom, entitled *Test di Autovalutazione della Competenza nella Comunicazione* (TACCom-IT), shows satisfactory cultural and linguistic equivalence in relation to the original instrument and is considered cross-culturally valid.

## INTRODUCTION

Communication is an exchange of information between two or more people, during which an alternation between interdependent processes of speaking and listening is expected; it is a generally spontaneous and automatic act, both in the social and professional spheres. As with most habitual activities, communication tends to rely only on natural skills, automatisms developed throughout life, instinct, and improvisation. Hence, the possibility of improving communication skills is not always considered or known, which can be done with the help of qualified professionals, providing greater quality in the interactions between the speaker and the listener^(1)^.

In most speaking situations, there is little awareness of the communicative attitude, which means that the speaker is unaware of the additional information that they may eventually be passing on to the listeners, beyond the linguistic content of the message, through nonverbal communication, vocal frequency and intensity, speech rate, pauses, and modulation. However, consciously or not, it is a fact that communication interferes with the quality of relationships built throughout life.

In a professional role that depends on good communication performance, a conscious development of this competence becomes necessary, given the importance and high degree of visibility involved in this context^(1,2)^. Career advancement does not directly depend on communication competence, but it is configured as a soft skill, a valued and expected socio-emotional skill, especially in high-level management positions, and can positively influence professional careers^(1,3)^.

There are several ways to communicate and to appropriate different communication styles, depending on the context and area of ​​professional activity. For efficient communication, it is important to consider mainly the objective of the message, the target audience, and the location or context of the communicative interaction^(4,5)^.

Communication competence can be improved in two ways: by modifying emotional aspects and by consciously controlling them. Modifying the emotional aspects related to this challenge may require coaching strategies or even psychological guidance. Speech-language-hearing (SLH) pathologists generally work on communication development, using two main approaches, often combined, based on language or the physical aspects of voice and speech. Work on language focuses on a more theoretical approach, starting from the organization of thought, issues of discourse construction and argumentation, while work based on voice and speech aspects is centered on techniques for controlling and stabilizing these components^(1)^, as well as conveying security and credibility.

To establish a starting point for the program to develop this competence and to monitor the evolution of the intervention, it is important to have an instrument capable of evaluating it. There is no specific test or questionnaire for this purpose in Italian. Hence, it is important to validate the Self-Assessment of Communication Competence (SACCom) for Italian to provide SLH pathologists with an instrument to assess communication competence in that language. SACCom, developed in Brazilian Portuguese, was created by Mara Behlau^(6)^ in 2002 and validated in 2022^(7)^.

SACCom^(7)^ consists of 19 items that assist in the self-assessment of communication behavior and includes nine aspects more directed to speaking and 10 aspects more directed to listening. As a self-assessment instrument, SACCom does not necessarily reflect the other person's perception of the speaker, but it is an important self-reflection strategy. The instrument can be used for different purposes, namely: evaluating communication efficiency and directing action towards the development of specific aspects of communication, whether they are formal (voice and speech), attitudinal (e.g., paying genuine attention to what is said), language (e.g., responding directly to what is asked), or listening (e.g., remembering important elements said by the interlocutor)^(1-7)^. It can also be a resource for comparing the responses given by self-assessment with the responses marked by third parties about the respondent^(8)^ to verify the difference between the speaker's self-perception and the real impact of communication on the listener.

The cross-cultural validation of assessment and self-assessment instruments is a relevant strategy for expanding the use of standardized measures in different linguistic and cultural contexts. In addition to favoring international scientific exchange, it allows the use of previously developed and theoretically consolidated instruments, avoiding the duplication of efforts in the construction of new measures. However, cross-cultural adaptation requires methodological rigor to ensure semantic, idiomatic, conceptual, and cultural equivalence between the original and the adapted version^(9)^. In the case of SACCom, its adaptation to the Italian context is justified by the absence of equivalent instruments that specifically and systematically assess communicative competence within the scope of SLH practice and professional performance. The availability of a culturally appropriate version expands the possibilities for clinical assessment, monitoring interventions, and research development in the area, also allowing international comparisons and the production of multicenter evidence.

Therefore, this study aimed to perform the cross-cultural validation of the SACCom for Italian.

## METHODS

This cross-sectional research was approved by the Research Ethics Committee of the Federal University of Sergipe (opinion number 4788134). The volunteers who agreed to participate in the study signed an informed consent form. The authors of SACCom authorized the cross-cultural validation study of the instrument for Italian.

The method followed the recommendations of the Consensus-based Standards for the Selection of Health Measurement Instruments (COSMIN)^(10)^, which was chosen as a methodological reference for having been developed and applied in the context of SLH practice, with direct implications for functional assessment, clinical decision-making, intervention planning, and results monitoring. In this framework, SACCom is conceived as a measure of communicative functioning, in the dimension of human performance related to social and professional participation, in accordance with the International Classification of Functioning, Disability and Health (ICF). Thus, although the instrument assesses communicative competencies of a behavioral nature, the construct is operationalized as a functional outcome related to health, which justifies the adoption of COSMIN to evaluate its measurement properties.

**Figure 1:**
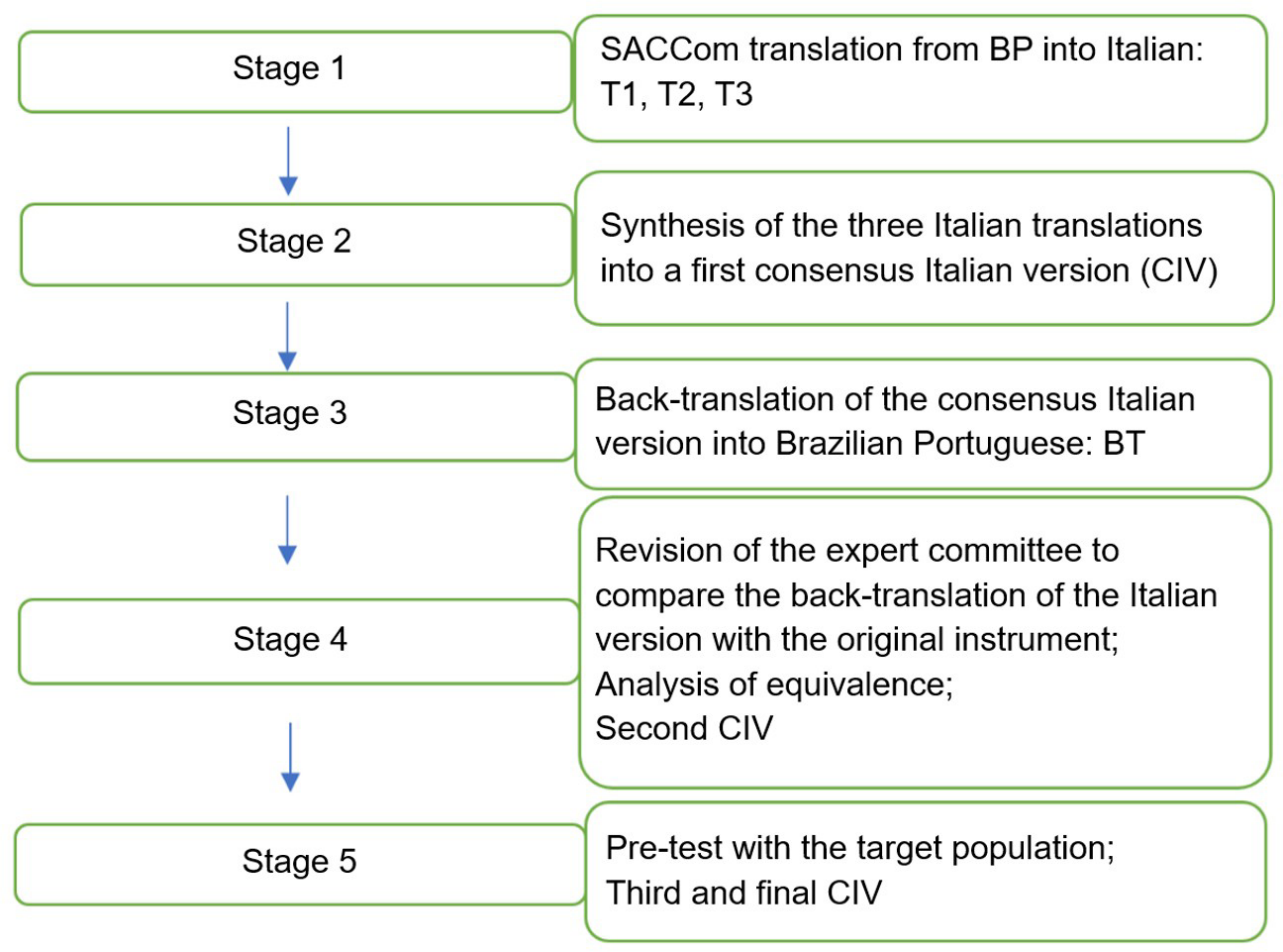
Flowchart of the cross-cultural validation process of SACCom for Italian (TACCom-IT) according to COSMIN recommendations

The cross-cultural validation stages were organized as follows:

- Stage 1: translation of the SACCom from Brazilian Portuguese to Italian, carried out by three bilingual SLH pathologists (indicated in Figure 1 and Chart 1 as T1, T2, T3); 2: synthesis of the three translated versions in Italian into a first consensus version, conducted by one of the authors of the study and reviewed by the co-authors; 3: back-translation of the first consensus version in Italian into Brazilian Portuguese, carried out by a fourth bilingual SLH pathologist (indicated in Figure 1 and Chart 1 as BT); 4: review by the expert committee for the comparison of the back-translation of the first version from Italian to the original, and analysis of semantic, conceptual, idiomatic, experiential, cultural, and operational equivalence; and 5, pre-test stage with the target population.

**Caption:** BP = Brazilian Portuguese, T1 = Portuguese-Italian translator 1; T2 = Portuguese-Italian translator 2; T3 = Portuguese-Italian translator 3; CIV = consensus Italian version; BT = back-translation from Italian to Brazilian Portuguese

**Chart 1:** SACCom cross-cultural validation process for Italian (TACCom-IT)

Original version	Translation	Synthesis	Back-translation	Consensus
Title				
Teste de Autoavaliação da Competência na Comunicação: TACCom	T1. Questionario di autovalutazione della competenza comunicativa (TACCom)	Test di autovalutazione della competenza comunicativa (TACCom)	Teste de autoavaliação da competência comunicativa (TACCom)	Test di Autovalutazione della Competenza nella Comunicazione (TACCom-IT)
	T2. Test di autovalutazione della capacità comunicativa (TACCom)			
	T3. Test di autovalutazione della competenza comunicativa (TACCom)			
Title/instructions				
As perguntas a seguir referem-se a diversos aspectos de fala e escuta. Leia atentamente cada uma delas e assinale se você tem ou não esses comportamentos de comunicação.	T1: Le seguenti domande si riferiscono a diversi aspetti del linguaggio e della percezione uditiva. Legga attentamente ognuna di essa e risponda in modo positivo o negativo se riconosce in lei questi comportamenti comunicativi	Le seguenti domande si riferiscono a diversi aspetti del parlato e dell’ascolto. Legga attentamente ciascuna domanda e scriva se ha o meno questi comportamenti comunicativi.	BT: As perguntas a seguir referem-se a diferentes aspectos da fala e da audição. Leia cada pergunta com atenção e anote se você tem ou não esses comportamentos comunicativos	Le seguenti domande si riferiscono a diversi aspetti del parlato e dell’ascolto. Legga attentamente ciascuna domanda e scriva se ha o meno questi comportamenti comunicativi.
	T2: Le seguenti domande si riferiscono a diversi aspetti del parlare e dell’ascolto. Leggi attentamente ogni domanda e segnali se hai o meno questi comportamenti comunicativi.			
	T3: Le domande che seguono si riferiscono a diversi aspetti del parlato e dell’ascolto. Legga attentamente ciascuna domanda e scriva se assume o meno questi comportamenti comunicativi.			
Answer key				
Sim	T1: sì	sì	R: sim	sì
	T2: sì			
	T3: si			
Não	T1: no	no	R: não	no
	T2: no			
	T3: no			
Items				
1. Você consegue captar e manter a atenção do ouvinte?	T1: Riesce a catturare e mantenere l’attenzione di chi la ascolta?	Riesce a catturare e mantenere l’attenzione di chi la ascolta?	R: Você consegue capturar e manter a atenção de quem está te escutando?	Riesce a catturare e mantenere l’attenzione di chi la ascolta?
	T2: Riesci catturare e mantenere l'attenzione dell'ascoltatore?			
	T3: Riesce a catturare e mantenere l’attenzione dell’ascoltatore?	*l’ascoltatore è troppo formale, è più frequente dire di chi ascolta.		
2. Sua voz é boa e expressiva?	T1: La sua voce è bella ed espressiva?	La sua voce è bella ed espressiva?	R: A tua voz é bonita e expressiva?	La sua voce è buona ed espressiva?
	T2: La tua voce è buona ed espressiva?			
	T3: Ritiene di avere una voce efficace?			
3. Você fala claro, com boa dicção?	T1: Parla bene, con una buona dizione?	Parla in modo chiaro, con una buona dizione?	R: Você fala de maneira clara, com uma boa dicção?	Parla in modo chiaro, con una buona dizione?
	T2: Parli chiaramente, con una buona dizione?			
	T3: Parla in modo chiaro, con una buona dizione?			
4. Você acha fácil influenciar os outros com sua comunicação?	T1: Pensa che sia facile influenzare gli altri attraverso il suo modo di comunicare?	Considera facile influenzare gli altri attraverso il suo modo di comunicare?	R: Você acha fácil influenciar outras pessoas através de sua maneira de comunicar?	Considera facile influenzare gli altri attraverso il suo modo di comunicare?
	T2: Trovi facile influenzare gli altri con la tua comunicazione?			
	T3: Considera facile influenzare gli altri attraverso il suo modo di comunicare?			
5. As pessoas lembram-se do que você disse?	T1: Le persone si ricordano di quello che aveva detto loro?	Le persone si ricordano di ciò che ha detto?	R: As pessoas se lembram do que você tem dito?	Le persone si ricordano di ciò che ha detto?
	T2: Le persone si ricordano ciò che hai detto?			
	T3: Le persone ricordano ciò che ha detto?			
6. Os outros deixam você falar, sem interrompê-lo?	T1: Gli altri la lasciano parlare, senza interromperla?	Gli altri la lasciano parlare senza interromperla?	R: Os outros permitem que você fale sem interrompê-lo?	Gli altri la lasciano parlare senza interromperla?
	T2: Gli altri ti lasciano parlare senza interromperti?			
	T3: Gli altri la lasciano parlare, senza interromperla?			
7. Você aproveita as oportunidades de comunicação?	T1: Approfitta delle opportunità comunicative?	Usufruisce/Sfrutta le opportunità di comunicazione che le si presentano?	R: Você utiliza / aproveita as oportunidades de comunicação que se apresentam?	Approfitta delle opportunità di comunicazione che le si presentano?
	T2: Usufruisci delle opportunità di comunicazione?			
	T3: Sfrutta le opportunità di comunicazione che le si presentano?			
8. Os outros aceitam suas sugestões, críticas ou *feedback* (opinião sua sobre os outros)?	T1: Gli altri accettano i suoi consigli, le sue critiche o i suoi feedback (sue opinioni sugli altri)	Gli altri accettano i suoi suggerimenti, le sue critiche o i suoi feedback/riscontri (ovvero la sua opinione sugli altri)?	R: Os outros aceitam suas sugestões, suas críticas ou seus feedback (ou seja, a sua opinião sobre os outros)?	Gli altri accettano i suoi suggerimenti, le sue critiche o i suoi feedback (ovvero la sua opinione sugli altri)?
	T2: Gli altri accettano i tuoi suggerimenti, critiche o feedback (la tua opinione sugli altri)?			
	T3: Gli altri accettano i suoi suggerimenti, le sue critiche o i suoi feedback (ovvero la sua opinione sugli altri)?			
9. Você usa a comunicação como parte do seu *marketing* pessoal?	T1: Utilizza la sua voce come parte del suo marketing personale?	Usa la comunicazione come parte del suo marketing personale?	R: Você usa a comunicação como parte do seu marketing pessoal?	Usa la comunicazione come parte del suo marketing personale?
	T2: Usi la comunicazione come parte del tuo marketing personale?			
	T3: Usa la comunicazione come parte del suo marketing personale?			
10. Você deixa o outro falar sem interrompê-lo?	T1: Lascia che gli altri parlino senza interromperli?	Lascia parlare gli altri senza interromperli?	R: Você deixa os outros falarem sem interrompê-los?	Lascia parlare gli altri senza interromperli?
	T2: Lasci parlare qualcun altro senza interromperlo?			
	T3: Lascia parlare gli altri senza interromperli?			
11. Você presta atenção na mensagem verbal e não verbal do que é dito (voz, linguagem corporal e gestos)?	T1: Presta attenzione al messaggio verbale e non verbale di quello che viene detto (voce, linguaggio corporeo e gestualità)?	Presta attenzione al messaggio verbale e non verbale di quello che viene detto (voce, linguaggio del corpo e gesti)?	R: Você presta atenção na mensagem verbal e não verbal do que está sendo dito (voz, linguagem do corpo e gestos)?	Presta attenzione al messaggio verbale e non verbale di quello che viene detto (voce, linguaggio del corpo e gestualità)?
	T2: Presti attenzione al messaggio verbale e non verbale di ciò che viene detto (voce, linguaggio del corpo e gesti)?			
	T3: Presta attenzione ai messaggi verbali e non verbali di ciò che viene detto (voce, linguaggio del corpo e gesti)?			
12. Você assume o que diz?	T1: Crede in ciò che dice?	Lei si prende la responsabilità di quello che dice?	R: Você assume a responsabilidade daquilo que diz?	Si prende la responsabilità di ciò che dice?
	T2: Prendi la responsabilità per quello che dici?			
	T3: Lei si riconosce in ciò che dice?			
13. Você focaliza a atenção na pessoa interlocutora (evitando ouvir conversas paralelas)?	T1: Focalizza l’attenzione sul suo interlocutore (evitando di ascoltare conversazioni parallele)?	Focalizza l’attenzione sull’interlocutore (evitando di ascoltare conversazioni parallele)?	R: Você focaliza a atenção no interlocutor (evitando de ouvir conversas paralelas)?	Focalizza l’attenzione sull’interlocutore (evitando di ascoltare conversazioni parallele)?
	T2: Focalizzi l'attenzione sull'interlocutore (evitando di ascoltare conversazioni secondarie)?			
	T3: Focalizza l’attenzione sull’interlocutore (evitando di ascoltare conversazioni parallele)?			
14. Você mantém a atenção no discurso da outra pessoa (evitando distrair-se com seus próprios pensamentos)?	T1: Riesce a mantenere l’attenzione su ciò che le dice l’altra persona (evitando che i suoi pensieri la distraggano)?	Mantiene l’attenzione sul discorso dell’altra persona (evitando di distrarsi con i propri pensieri)?	R: Você mantém a atenção no discurso da outra pessoa (evitando de se distrair com seus próprios pensamentos)?	Mantiene l’attenzione su ciò che le dicono (evitando di distrarsi con i propri pensieri)?
	T2: Concentri l’attenzione sul discorso dell'altra persona (evitando di distrarti con i tuoi propri pensieri)?			
	T3: Mantiene l’attenzione sul discorso dell’altra persona (evitando di distrarsi con i propri pensieri)?			
15. Você responde diretamente ao que é perguntado (sem rodeios)?	T1: Risponde direttamente a ciò che le viene chiesto (senza giri di parole)?	Risponde direttamente a ciò che le viene chiesto (senza giri di parole)?	R: Você responde diretamente ao que te vem perguntado (sem meias palavras)?	Risponde direttamente a ciò che le viene chiesto (senza giri di parole)?
	T2: Rispondi direttamente a quello che ti vieni chiesto (senza mezzi termini)?			
	T3: Risponde direttamente a ciò che le viene chiesto (senza giri di parole)?			
16. Você mostra interesse no que está sendo dito, por meio do olhar, postura ou sinais de apoio e aprovação?	T1: Dimostra interesse per ciò che sta per essere detto, attraverso lo sguardo, la postura o segnali di sostegno e approvazione?	Mostra interesse per ciò che viene detto, tramite lo sguardo, la postura o segnali di supporto ed approvazione?	R: Você mostra interesse pelo que está sendo dito, por meio do olhar, da postura ou de sinais de suporte e aprovação?	Mostra interesse per ciò che viene detto, tramite lo sguardo, la postura o segnali di supporto ed approvazione?
	T2: Mostri interesse per ciò che viene detto, attraverso lo sguardo, la postura o segni di sostegno e di approvazione?			
	T3: Mostra interesse in ciò che viene detto, tramite lo sguardo, la postura o segnali di supporto ed approvazione?			
17. Você repete os pontos importantes do que foi dito para se certificar que compreendeu bem?	T1: Ripete i punti chiavi di ciò che è stato detto per essere certo di aver capito bene?	Ripete i punti importanti di ciò che è stato detto per essere sicuro di aver capito bene?	R: Você repete os pontos importantes do que foi dito para estar seguro de ter entendido bem?	Ripete i punti importanti di ciò che le dicono per essere sicuro di aver capito bene?
	T2: Ripeti i punti importanti di ciò che è stato detto per essere sicuri di aver capito bene?			
	T3: Ripete ed analizza i punti più importanti di ciò che le è stato detto per assicurarsi di aver compreso bene?			
18. Você procura memorizar fatos importantes e características da pessoa interlocutora?	T1: Cerca di memorizzare fatti importanti e caratteristiche salienti del suo interlocutore?	Cerca di memorizzare fatti importanti e caratteristiche del suo interlocutore?	R: Você tenta memorizar fatos importantes e características de seu interlocutor?	Cerca di memorizzare fatti importanti e caratteristiche del suo interlocutore?
	T2: Cerchi di memorizzare fatti importanti e caratteristiche dell'interlocutore?			
	T3: Cerca di memorizzare eventi importati e caratteristiche del suo interlocutore?			
19. Você recebe bem críticas, sugestões ou *feedback* (opinião dos outros sobre você)?	T1: Riceve critiche, suggerimenti, feedback (opinione degli altri su di voi)?	Accoglie bene le critiche, i suggerimenti o i feedback (ovvero le opinioni degli altri su di lei)?	R: Você aceita bem as críticas, as sugestões ou os feedback (ou seja, as opiniões dos outros sobre você)?	Accetta le critiche, i suggerimenti o i feedback (ovvero le opinioni degli altri su di lei)?
	T2: Accetti bene critiche, suggerimenti o feedback (l'opinione degli altri su di te)?			
	T3: Accoglie bene le critiche, i suggerimenti o i feedback (ovvero le opinioni degli altri su di lei)?			

**Caption:** BP = Brazilian Portuguese, IT = Italian, T1 = Portuguese-Italian translator 1; T2 = Portuguese-Italian translator 2; T3 = Portuguese-Italian translator 3; BT = back-translation from Italian to Brazilian Portuguese; CIV = consensus Italian version.

The sample for the first four stages consisted of eight SLH pathologists (six bilingual and two non-bilingual), specialists in the construct. Four of them were part of the study's author team and participated in stages 2 and 4. The sample for the fifth stage consisted of 101 Italians, born and residing in Italy, who use their voice and oral communication in their profession. Information about the profession, self-reported by the study participants in the data collection form, was used to classify the occupational voice users. The study also used the COSMIN recommendation^(10)^ of a minimum sample size of 30 individuals for the pre-test.

The eligibility criteria for the four SLH pathologists responsible for the translation and back-translation stages were being a bilingual Italian-Brazilian SLH pathologist with linguistic competence in both languages and knowing the construct and culture of both countries (Italy and Brazil). Participants were selected by convenience and through curriculum analysis, evaluating professional experience in the field of voice and experience in both countries, through activities related to SLH practice, and a telephone interview conducted in both languages. For the fifth stage (pre-test), Italian professionals from various fields whose voice and oral communication are essential to their profession, aged 18 and over, currently residing in Italy, were recruited through an invitation disseminated on social media. Unemployed individuals and/or those not working in areas where voice plays a central role were excluded. By clicking on the link in the invitation, participants directly accessed the eligibility questionnaire, available on Google Drive, at the end of which they signed an informed consent form. Of the 198 volunteer participants, 101 were selected after meeting the eligibility criteria.

In the first stage, three SLH pathologists translated the SACCom into Italian, encompassing its title, instructions, items, and answer key options. They respected the format, content, and concepts of the original version, although with adaptations of cultural and linguistic aspects concerning Italian. This stage was independent and blind, without any interaction between the participants.

In the second stage (synthesis stage), three of the authors of this article compared and superimposed the three translated versions, deciding on the best translation by consensus, considering cultural and semantic equivalence. The first Italian version of the SACCom was thus constructed and validated by all the authors of this article.

The third stage consisted of sending the consensus version in Italian to a fourth SLH pathologist, who had not participated in the previous stages and who had not had access to the original version of the instrument, to back-translate it into Brazilian Portuguese, its original language.

In the fourth stage, an expert committee was formed with four SLH pathologists, one specializing in methodology and three specializing in voice, with at least 5 years since graduation, including authors and non-authors of this study. The committee compared the back-translated version with the original instrument, from which only lexical differences emerged, as well as a conceptual difference in the title. After analysis, they decided by consensus on the changes necessary for the final version, with semantic, conceptual, idiomatic, experiential, cultural, and operational equivalence. The Italian version of the SACCom was named *Test di Autovalutazione della Competenza nella Comunicazione* (TACCom-IT). Figure 1 and Chart 1 illustrate the four phases of the cross-cultural validation process of the SACCom for the TACCom-IT.

In the pre-test stage, 101 Italians responded to the TACCom-IT. Their mean age was 40 years, with 22 men and 79 women, from various areas that essentially require the use of their voice and oral communication. The professional categories were entrepreneurs, lawyers, managers, sales representatives, teachers, and health professionals.

In this phase, all participants responded to the TACCom-IT. The original SACCom^(7)^ is a self-assessment instrument of communication competence, with 19 yes/no questions related to speaking and listening skills in interpersonal communication. Statistical calculation of the responses is carried out using a specific spreadsheet, given that the validation was carried out by Item Response Theory^(9)^. For this pre-test phase, the option “not applicable” was added to the dichotomous response key (yes/no), translated as “*la domanda non è chiara*”. This option should be checked if the participants did not understand the item, or if it did not apply to Italian culture. In addition, a space for comments was added in case the participant answered "not applicable," although filling it out was not mandatory. The number of participants who responded to the options in the dichotomous response key was compared to the number of those who responded to the "not applicable" option using the two-proportion z-test. The analysis was performed using IBM SPSS Statistics 29.0.

## RESULTS

In the second stage, which involved synthesizing the three Italian translations into a first version, it was possible to reach a consensus regarding lexical choices among at least two SLH pathologists for the title, instructions, answer key options, and 13 items. However, there was no consensus at the lexical level in items 2, 4, 7, 12, 17, or 19; therefore, one of the three translations was chosen.

In the back-translation, there was no conceptual agreement regarding the title or instructions, but there was agreement regarding the answer key options in items 8, 9, 10, 13, and 14. As for items 1, 2, 3, 4, 5, 6, 11, 15, 16, 17, 18, and 19, there was no lexical agreement. Also, the fourth SLH pathologist added a phrase in two items 7, 12, respectively, "that appear" and "take responsibility for what you say."

In the fourth stage, the expert committee decided to add the acronym IT to the title (to refer to the Italian version of SACCom). It was also necessary to modify an expression in the title of the Italian version, from *Test di Autovalutazione della Competenza Comunicativa* to *Test di Autovalutazione della Competenza nella Comunicazione* ***,*** to meet semantic equivalence criteria. The translation of the instructions was maintained as in the synthesis version. In items 2, 7, and 19, one word per item was corrected to meet the cultural equivalence criteria. In the other items, satisfactory cultural and linguistic equivalences were obtained.

After concluding the stage based on the modifications and adjustments of the expert committee, a second cross-culturally validated version of the TACCom-IT was constructed. All the data described throughout the four initial stages are shown in Chart 1.

In the pre-test stage (Table 1), it was not possible to statistically analyze items 3, 10, and 12, since all participants responded with one option from the instrument's usual response key (yes/no). For all other items, there was a significantly higher proportion of participants who responded with options from the instrument's usual response key (yes/no), compared to the proportion of participants who responded, “not applicable” (p < 0.001 for all).

**Table 1:** Comparison of the proportion of participants who responded using the usual response keys and those who responded using the “non-applicable” response key

Item	Yes/ No		Not applicable		p-value
	n	%	n	%	
1. Riesce a catturare e mantenere l’attenzione di chi la ascolta?	100	99.01	1	0.99	<0.001*
2. La sua voce è buona ed espressiva?	97	96.04	4	3.96	<0.001*
3. Parla in modo chiaro, con una buona dizione?	101	100	0	0	
4. Considera facile influenzare gli altri attraverso il suo modo di comunicare?	99	98.02	2	1.98	<0.001*
5. Le persone si ricordano di ciò che ha detto?	95	94.06	6	5.94	<0.001*
6. Gli altri la lasciano parlare senza interromperla?	98	97.03	3	2.97	<0.001*
7. Approfitta delle opportunità di comunicazione che le si presentano?	92	91.09	9	8.91	<0.001*
8. Gli altri accettano i suoi suggerimenti, le sue critiche o i suoi feedback (ovvero la sua opinione sugli altri)?	92	91.09	9	8.91	<0.001*
9. Usa la comunicazione come parte del suo marketing personale?	93	92.08	8	7.92	<0.001*
10. Lascia parlare gli altri senza interromperli?	101	100	0	0	
11. Presta attenzione al messaggio verbale e non verbale di quello che viene detto (voce, linguaggio del corpo e gestualità)?	100	99.01	1	0.99	<0.001*
12. Si prende la responsabilità di ciò che dice?	101	100	0	0	
13. Focalizza l’attenzione sull’interlocutore (evitando di ascoltare conversazioni parallele)?	98	97.03	3	2.97	<0.001*
14. Mantiene l’attenzione su ciò che le dicono (evitando di distrarsi con i propri pensieri)?	98	97.03	3	2.97	<0.001*
15. Risponde direttamente a ciò che le viene chiesto (senza giri di parole)?	98	97.03	3	2.97	<0.001*
16. Mostra interesse per ciò che viene detto, tramite lo sguardo, la postura o segnali di supporto ed approvazione?	100	99.01	1	0.99	<0.001*
17. Ripete i punti importanti di ciò che le dicono per essere sicuro di aver capito bene?	99	98.02	2	1.98	<0.001*
18. Cerca di memorizzare fatti importanti e caratteristiche del suo interlocutore?	100	99.01	1	0.99	<0.001*
19. Accetta le critiche, i suggerimenti o i feedback (ovvero le opinioni degli altri su di lei)?	100	99.01	1	0.99	<0.001^*^

* *P-values obtained using the* Two-proportion z-test

**Caption:** n = absolute frequency; % = relative percentage frequency

Regarding interpretability, some pre-test participants selected the option “not applicable” (i.e., “*la domanda non è chiara*”), justifying, in the space available for comments, that the choice did not refer to the clarity or cultural appropriateness of the item, but to the difficulty of marking a dichotomous answer, an absolute “no” or “yes”, because some communication behaviors may vary according to the interlocutor and the context. After analyzing these comments, the expert committee decided in the interpretability stage to maintain the instructions of the consensus version, adding, however, a sentence that paraphrases the instructions, ensuring cultural equivalence. Thus, the final version of the TACCom-IT was consolidated (Appendix A).

## DISCUSSION

In today's dynamic and competitive job market, several soft skills, or socio-emotional skills, contribute to the success of organizations. Among them is the ability to communicate effectively. Its importance is so great that some authors suggest using the expression "essential skill" instead of "soft skill" to refer to this competence, which is highly sought after in senior positions in organizations, and whose absence can negatively influence the chance of a promotion or even contribute to the dismissal of a professional^(8)^.

Communicative competence, in the world of organizations, is not always a requirement in the initial phase of career development, but it becomes an essential resource and a prerequisite when moving from an operational role to management positions. At this point, conceptual communication skills may be required, such as the cognitive ability to visualize, understand, and coordinate all the activities and interests of organizations in a system, understanding their interrelationships^(11,12)^.

A professional who communicates in a thoughtful, planned, structured, prepared, and assertive manner can generate considerable psychological impact on their listener, giving them the impression of professionalism and security, which reflects clarity of reasoning and thought and mastery of the rules of the organizational world. Therefore, much has been sought in developing this competence in leadership positions.

For communication to be developed assertively and consciously, it is necessary to have assessment tools that help professionals understand which skills already exist, which need to be improved, and which need to be acquired. There is no questionnaire for analyzing the perception of competence in communication in Italian, which justifies the cross-cultural validation for this language of the SACCom, a self-assessment instrument of communication competence validated in Brazilian Portuguese.

This cross-cultural validation found some lexical and conceptual disagreements during the translation and back-translation process of TACCom-IT. These data corroborate the importance of following well-structured steps in the cross-cultural validation process of an instrument and confirm that a simple literal translation is by no means sufficient, as it ignores and disregards cultural complexity^(13)^.

Semantic and conceptual equivalence indicated the need for adjustments to the title and instructions, ensuring the same semantic weight as the original version. Regarding the title, the discussion determined the best expression, in Italian, to translate the phrase "competence in communication." This concept can be translated in several ways: *capacità comunicativa, capacità di comunicazione, competenza comunicativa, abilità comunicative, competenza nella comunicazione*. Among them, *competenza comunicativa* (communicative competence) was chosen in the synthesis phase of the study. All options refer to both concepts: linguistic or grammatical competence and speaking and listening competence in communication. However, in Portuguese, communicative competence refers exclusively to linguistic or grammatical competence to produce sentences that are understood not only as products of a given language but also coherent with the speaker's intention in a given circumstance. It also involves textual competence, seen as the user's ability, in situations of communicative interaction, to produce, understand, transform, and classify texts that are appropriate to the intended communicative interaction, following regularities and principles of text organization and construction and textual functioning, since texts are the unit of language in use. In the phase of expert committee analysis, it was decided by consensus to modify the expression to *competenza nella comunicazione*, to ensure semantic and conceptual equivalence between TACCom-IT and SACCom.

In this same phase, word adjustments were made in items 2, 4, 7, 12, 17, and 19 so that the meaning reflected that of the SACCom, and that the synonym culturally respected the Italian context^(14)^.

In the pre-test phase of the TACCom-IT, there was a significantly higher proportion of participants who responded with options from the instrument's usual response key, when compared to the “not applicable” option. The items in which there was no difference were those for which all participants responded with the usual options from the instrument's response key. Thus, there was no need to modify or eliminate items.

Regarding interpretability, some adjustments were necessary, considering the comments left by the participants in the pre-test phase. Interpretability refers to the degree to which qualitative meaning can be attributed to the quantitative scores of an instrument or changes in quantitative scores^(10)^. A distinct characteristic was observed in the Italian population, where participants had difficulty choosing between the options in the original response key (“yes” and “no”). To resolve this discomfort, it was decided to maintain the original response key and the instructions from the consensus version and include a sentence paraphrasing the TACCom-IT instructions. The initial instruction, “*Le seguenti domande si riferiscono a diversi aspetti del parlato e dell’ascolto. Legga attentamente ciascuna domanda e scriva se ha o meno questi comportamenti comunicativi*” (“The following questions refer to different aspects of speaking and listening. Read each question carefully and indicate whether you exhibit these communicative behaviors”) was adjusted to, *“Le seguenti domande si riferiscono a diversi aspetti del parlato e dell’ascolto. Legga attentamente ciascuna domanda e scriva se ha o meno questi comportamenti comunicativi. Risponda SI, se questi comportamenti si compiono/sono presenti la maggior parte delle volte e risponda NO, se questi comportamenti non sono presenti la maggior parte delle volte”* (“The following questions refer to different aspects of speaking and listening. Read each question carefully and indicate whether you exhibit these communicative behaviors. Answer YES if these behaviors occur/are present most of the time and answer NO if these behaviors are not present most of the time”). This adjustment aimed to ensure the good interpretability of the TACCom-IT and to guide participants in choosing the response key that best reflects real communicative behavior^(15)^.

Despite the satisfactory results in cross-cultural validation, it will be necessary to demonstrate the other psychometric properties of the instrument in the Italian population to ensure the validity, reliability, and responsiveness of the TACCom-IT, which should be done in future studies.

As a limitation, it should be noted that the sociodemographic description of the sample was restricted to their age, sex, and profession. The inclusion of additional information could enrich the characterization of the investigated group.

## CONCLUSION

The cross-culturally validated Italian version of the SACCom was named *Test di Autovalutazione della Competenza nella Comunicazione* (TACCom-IT) and maintained the instrument's original structure. However, in addition to adjustments to the usual cross-cultural validation processes, an adaptation of the test instructions was necessary to obtain all the required equivalences. The study presented satisfactory results regarding the cross-cultural validation of the TACCom-IT.

## Appendix A Final cross-culturally validated version of SACCom

TEST DI AUTOVALUTAZIONE DELLA COMPETENZA NELLA COMUNICAZIONE (TACCom-IT)

Nome: ______________________________________ Data: ___/___/____

Le seguenti domande si riferiscono a diversi aspetti del parlato e dell’ascolto. Legga attentamente ciascuna domanda e scriva se ha o meno questi comportamenti comunicativi. Risponda SI, se questi comportamenti si compiono/sono presenti la maggior parte delle volte e risponda NO, se questi comportamenti non sono presenti la maggior parte delle volte

**Table:**

1. Riesce a catturare e mantenere l’attenzione di chi la ascolta?	sì	no
2. La sua voce è buona ed espressiva?	sì	no
3. Parla in modo chiaro, con una buona dizione?	sì	no
4. Considera facile influenzare gli altri attraverso il suo modo di comunicare?	sì	no
5. Le persone si ricordano di ciò che ha detto?	sì	no
6. Gli altri la lasciano parlare senza interromperla?	sì	no
7. Approfitta delle opportunità di comunicazione che le si presentano?	sì	no
8. Gli altri accettano i suoi suggerimenti, le sue critiche o i suoi *feedback* (ovvero la sua opinione sugli altri)?	sì	no
9. Usa la comunicazione come parte del suo *marketing* personale?	sì	no
10. Lascia parlare gli altri senza interromperli?	sì	no
11. Presta attenzione al messaggio verbale e non verbale di quello che viene detto (voce, linguaggio del corpo e gestualità)?	sì	no
12. Si prende la responsabilità di ciò che dice?	sì	no
13. Focalizza l’attenzione sull’interlocutore (evitando di ascoltare conversazioni parallele)?	sì	no
14. Mantiene l’attenzione su ciò che le dicono (evitando di distrarsi con i propri pensieri)?	sì	no
15. Risponde direttamente a ciò che le viene chiesto (senza giri di parole)?	sì	no
16. Mostra interesse per ciò che viene detto, tramite lo sguardo, la postura o segnali di supporto ed approvazione?	sì	no
17. Ripete i punti importanti di ciò che le dicono per essere sicuro di aver capito bene?	sì	no
18. Cerca di memorizzare fatti importanti e caratteristiche del suo interlocutore?	sì	no
19. Accetta le critiche, i suggerimenti o i *feedback* (ovvero le opinioni degli altri su di lei)?	sì	no
