# Supplementary material for: Cross-cultural validation of the self-assessment of competence in communication – SACCom – to Italian
Source: Codas. 2026 Aug 3;38(5):e20250281. doi: 10.1590/2317-1782/e20250281en (PMC13445986; doi:10.1590/2317-1782/e20250281en)
Supplement: Translation

# Translation: Validação transcultural para o italiano do teste de autoavaliação da competência na comunicação: TACCom

**DOI:** 10.1590/2317-1782/e20250281pt

**Authors:** Aurora d’Apolito, Vanessa Veis Ribeiro, Glaucya Madazio, Mara Behlau

**Affiliations:** 1 Centro de Estudos da Voz – CEV - São Paulo (SP), Brasil.

**Keywords:** Protocolos Clínicos, Autoavaliação, Fonoaudiologia, Comunicação, Voz, Fala, Relações Interpessoais

## Abstract

**Objetivo:**

Realizar a validação transcultural do Teste de Autoavaliação da Competência na Comunicação (TACCom) para a língua italiana.

**Método:**

O procedimento de validação transcultural seguiu as recomendações e diretrizes do COnsensus-based Standards for the selection of health Measurement INstruments (COSMIN) e constou de cinco etapas: 1) Tradução do TACCom do português brasileiro (PB) para o italiano, por três fonoaudiólogos bilíngues; 2) Elaboração da versão por consenso; 3) Retrotradução do italiano para o PB por um quarto fonoaudiólogo bilíngue; 4) Comparação da retrotradução com o instrumento original e elaboração da versão final em italiano, denominada *Test di Autovalutazione della Competenza nella Comunicazione* (TACCom-IT); e 5) Aplicação do pré-teste do TACCom-IT em 101 indivíduos nativos italianos, que usam a voz e a comunicação oral como principal instrumento de trabalho. Nesta etapa, a opção “não aplicável” foi acrescentada na chave de respostas.

**Resultados:**

Na tradução, houve escolhas lexicais diferentes em relação ao título, às instruções e a sete itens. Na retrotradução, houve discordâncias conceituais no título e nas instruções, e lexicais em cinco itens. O comitê de especialistas realizou mudanças no título, nas instruções e em três itens. Na fase pré-teste, uma proporção significativamente maior de participantes respondeu com opções da chave de resposta habitual do instrumento para todos os itens.

**Conclusão:**

A versão adaptada do TACCom para o italiano, intitulada *Test di Autovalutazione della Competenza nella Comunicazione* (TACCom-IT), apresenta equivalência cultural e linguística satisfatória em relação ao instrumento original, sendo considerada válida transculturalmente.

## INTRODUÇÃO

A comunicação é uma troca de informações entre duas ou mais pessoas, durante a qual se espera uma alternância entre processos interdependentes de fala e escuta; é um ato geralmente espontâneo e automático, tanto na esfera social como na profissional. Tal como acontece com a maioria das atividades habituais, na comunicação tende-se a recorrer apenas às habilidades naturais, aos automatismos desenvolvidos ao longo da vida, ao instinto e à improvisação, e nem sempre se considera ou se conhece a possibilidade de aperfeiçoar a habilidade de competência na comunicação, o que pode ser feito com a ajuda de profissionais habilitados, propiciando maior qualidade nas interações entre o falante e o ouvinte^(1)^.

Na maioria das situações de fala tem-se pouca consciência sobre a atitude comunicativa, o que faz com que o falante não se dê conta das informações adicionais que possa eventualmente estar passando aos ouvintes, para além do conteúdo linguístico da mensagem, por meio tanto da comunicação não-verbal, como da frequência e intensidade vocal, velocidade da fala, pausas e modulação. Porém, de forma consciente ou não, é um fato que a comunicação interfere na qualidade dos relacionamentos construídos ao longo da vida.

Em uma atuação profissional que dependa de uma boa performance na comunicação, torna-se necessário um desenvolvimento consciente dessa competência, dada a importância e o alto grau de visibilidade envolvidos nesse contexto^(1,2)^. A evolução de uma carreira não depende diretamente da competência na comunicação, mas esta se configura como uma *soft skill*, uma habilidade socioemocional valorizada e esperada, principalmente em cargos de alta gestão, podendo influenciar positivamente na carreira profissional^(1,3)^.

Existem várias formas de se comunicar e de se apropriar dos diferentes estilos de comunicação, dependendo do contexto e da área de atuação profissional. Para uma comunicação eficiente, é importante considerar principalmente o objetivo da mensagem, o público-alvo, e o local ou o contexto da interação comunicativa^(4,5)^.

Há duas vias principais de trabalho para a melhoria da competência na comunicação: a da modificação dos aspectos emocionais e a do controle consciente dela. A modificação dos aspectos emocionais relacionados a esse desafio pode requerer estratégias de *coaching* ou até mesmo orientação psicológica. A atuação sobre o desenvolvimento da comunicação geralmente é realizada por fonoaudiólogos, por meio de dois acessos principais, muitas vezes usados de forma associada, com base na linguagem ou nos aspectos físicos da voz e da fala. O trabalho sobre a linguagem tem foco em uma abordagem mais teórica, partindo-se da organização do pensamento, das questões da construção do discurso e da elaboração da argumentação, enquanto a atuação com base nos aspectos da voz e da fala está centrada em técnicas para o controle e estabilidade desses componentes^(1)^, além da transmissão de segurança e credibilidade.

Para estabelecer um ponto de partida para o programa de desenvolvimento dessa competência e monitorar a evolução da intervenção, é importante dispor de um instrumento capaz de avaliá-la. Na língua italiana, não há um teste ou questionário específico para tal propósito. Com o intuito de proporcionar aos fonoaudiólogos italianos um instrumento de avaliação da competência na comunicação, considera-se importante validar para o italiano o Teste de Autoavaliação da Competência na Comunicação – TACCom. O TACCom, desenvolvido em português brasileiro, foi elaborado por Mara Behlau^(6)^, em 2002, e validado em 2022^(7)^.

O TACCom^(7)^ é composto por 19 itens que auxiliam na autoavaliação do comportamento na comunicação e inclui 9 aspectos mais direcionados à fala e 10 aspectos mais direcionados à escuta. Por ser um instrumento de autoavaliação, o TACCom não reflete, necessariamente, a percepção do outro sobre o falante, mas é uma estratégia importante de autorreflexão. O instrumento pode ser empregado para diferentes finalidades, a saber: a avaliação da eficiência na comunicação e o direcionamento da ação para o desenvolvimento de aspectos específicos da comunicação, sejam eles: formais, de voz e fala; atitudinais, como prestar atenção genuína ao que se diz; de linguagem, como responder diretamente ao que se é perguntado; ou de escuta, como se lembrar de elementos importantes ditos pelo interlocutor^(1-7)^. Além disso, ele também pode ser um recurso de comparação das respostas dadas por autoavaliação com as respostas assinaladas por terceiros sobre o respondente^(8)^, a fim de verificar a diferença entre a autopercepção do falante e o real impacto da comunicação sobre o ouvinte.

A validação transcultural de instrumentos de avaliação e autoavaliação constitui estratégia relevante para a ampliação do uso de medidas padronizadas em diferentes contextos linguísticos e culturais. Além de favorecer o intercâmbio científico internacional, possibilita a utilização de instrumentos previamente desenvolvidos e teoricamente consolidados, evitando a duplicação de esforços na construção de novas medidas. No entanto, a adaptação transcultural exige rigor metodológico para assegurar equivalências semântica, idiomática, conceitual e cultural entre a versão original e a versão adaptada^(9)^. No caso do TACCom, sua adaptação para o contexto italiano justifica-se pela ausência de instrumentos equivalentes que avaliem, de forma específica e sistematizada, a competência comunicativa no âmbito da prática fonoaudiológica e do desempenho profissional. A disponibilização de uma versão culturalmente adequada amplia as possibilidades de avaliação clínica, de monitoramento das intervenções e de desenvolvimento das pesquisas na área, além de permitir comparações internacionais e produção de evidências multicêntricas.

Sendo assim, o objetivo do presente estudo foi o de realizar a validação transcultural do Teste de Autoavaliação da Competência na Comunicação (TACCom) para a língua italiana.

## MÉTODO

A presente pesquisa tem delineamento transversal, e foi aprovada pelo Comitê de Ética em Pesquisa da Universidade Federal de Sergipe (parecer número 4788134). Os voluntários que concordaram em participar da pesquisa assinaram o Termo de Consentimento Livre e Esclarecido. Os autores do TACCom autorizaram o estudo de validação transcultural do instrumento para a língua italiana.

A metodologia adotada seguiu as recomendações do Consensus-based Standards for the Selection of Health Measurement INstruments (COSMIN)^(10)^. O COSMIN foi escolhido como referencial metodológico, por se tratar de um instrumento desenvolvido e aplicado no contexto da prática fonoaudiológica, com implicações diretas para a avaliação funcional, a tomada de decisão clínica, o planejamento de intervenção e o monitoramento de resultados. Nesse enquadramento, o TACCom é concebido como uma medida do funcionamento comunicativo, na dimensão do desempenho humano relacionada à participação social e profissional, em consonância com a Classificação Internacional de Funcionalidade, Incapacidade e Saúde (CIF). Assim, embora o instrumento avalie competências comunicativas de natureza comportamental, o construto é operacionalizado como um desfecho funcional relacionado à saúde, o que justifica a adoção do COSMIN para a avaliação de suas propriedades de medida.

As etapas da validação transcultural foram organizadas da seguinte forma:

- Etapa 1: tradução do TACCom do PB para o italiano, realizada por três fonoaudiólogos bilíngues (indicados na figura e no Quadro 1, como T1, T2, T3); 2: síntese das três versões traduzidas em italiano, em uma primeira versão consenso, conduzida por um dos autores do estudo e conferida pelos coautores; 3: retrotradução da primeira versão consenso em italiano para o PB, realizada por um quarto fonoaudiólogo bilíngue (indicada na figura e no Quadro 1 como R); 4: revisão do comitê de especialistas para a comparação da retrotradução da primeira versão do italiano para o original, e análise das equivalências semântica, conceitual, idiomática, experiencial, cultural e operacional; e 5, etapa de pré-teste com a população-alvo.

**Quadro 1:** Processo de validação transcultural do TACCom para o italiano (TACCom-IT)

Versão Original	Tradução	Síntese	Retrotradução	Consenso
Título				
Teste de Autoavaliação da Competência na Comunicação: TACCom	T1. Questionario di autovalutazione della competenza comunicativa (TACCom)	Test di autovalutazione della competenza comunicativa (TACCom)	Teste de autoavaliação da competência comunicativa (TACCom)	Test di Autovalutazione della Competenza nella Comunicazione (TACCom-IT)
	T2. Test di autovalutazione della capacità comunicativa (TACCom)			
	T3. Test di autovalutazione della competenza comunicativa (TACCom)			
Título/instruções				
As perguntas a seguir referem-se a diversos aspectos de fala e escuta. Leia atentamente cada uma delas e assinale se você tem ou não esses comportamentos de comunicação.	T1: Le seguenti domande si riferiscono a diversi aspetti del linguaggio e della percezione uditiva. Legga attentamente ognuna di essa e risponda in modo positivo o negativo se riconosce in lei questi comportamenti comunicativi	Le seguenti domande si riferiscono a diversi aspetti del parlato e dell’ascolto. Legga attentamente ciascuna domanda e scriva se ha o meno questi comportamenti comunicativi.	R: As perguntas a seguir referem-se a diferentes aspectos da fala e da audição. Leia cada pergunta com atenção e anote se você tem ou não esses comportamentos comunicativos	Le seguenti domande si riferiscono a diversi aspetti del parlato e dell’ascolto. Legga attentamente ciascuna domanda e scriva se ha o meno questi comportamenti comunicativi.
	T2: Le seguenti domande si riferiscono a diversi aspetti del parlare e dell’ascolto. Leggi attentamente ogni domanda e segnali se hai o meno questi comportamenti comunicativi.			
	T3: Le domande che seguono si riferiscono a diversi aspetti del parlato e dell’ascolto. Legga attentamente ciascuna domanda e scriva se assume o meno questi comportamenti comunicativi.			
Chave de resposta				
Sim	T1: sì	sì	R: sim	sì
	T2: sì			
	T3: si			
Não	T1: no	no	R: não	no
	T2: no			
	T3: no			
Itens				
1. Você consegue captar e manter a atenção do ouvinte?	T1: Riesce a catturare e mantenere l’attenzione di chi la ascolta?	Riesce a catturare e mantenere l’attenzione di chi la ascolta?	R: Você consegue capturar e manter a atenção de quem está te escutando?	Riesce a catturare e mantenere l’attenzione di chi la ascolta?
	T2: Riesci catturare e mantenere l'attenzione dell'ascoltatore?			
	T3: Riesce a catturare e mantenere l’attenzione dell’ascoltatore?	*l’ascoltatore è troppo formale, è più frequente dire di chi ascolta.		
2. Sua voz é boa e expressiva?	T1: La sua voce è bella ed espressiva?	La sua voce è bella ed espressiva?	R: A tua voz é bonita e expressiva?	La sua voce è buona ed espressiva?
	T2: La tua voce è buona ed espressiva?			
	T3: Ritiene di avere una voce efficace?			
3. Você fala claro, com boa dicção?	T1: Parla bene, con una buona dizione?	Parla in modo chiaro, con una buona dizione?	R: Você fala de maneira clara, com uma boa dicção?	Parla in modo chiaro, con una buona dizione?
	T2: Parli chiaramente, con una buona dizione?			
	T3: Parla in modo chiaro, con una buona dizione?			
4. Você acha fácil influenciar os outros com sua comunicação?	T1: Pensa che sia facile influenzare gli altri attraverso il suo modo di comunicare?	Considera facile influenzare gli altri attraverso il suo modo di comunicare?	R: Você acha fácil influenciar outras pessoas através de sua maneira de comunicar?	Considera facile influenzare gli altri attraverso il suo modo di comunicare?
	T2: Trovi facile influenzare gli altri con la tua comunicazione?			
	T3: Considera facile influenzare gli altri attraverso il suo modo di comunicare?			
5. As pessoas lembram-se do que você disse?	T1: Le persone si ricordano di quello che aveva detto loro?	Le persone si ricordano di ciò che ha detto?	R: As pessoas se lembram do que você tem dito?	Le persone si ricordano di ciò che ha detto?
	T2: Le persone si ricordano ciò che hai detto?			
	T3: Le persone ricordano ciò che ha detto?			
6. Os outros deixam você falar, sem interrompê-lo?	T1: Gli altri la lasciano parlare, senza interromperla?	Gli altri la lasciano parlare senza interromperla?	R: Os outros permitem que você fale sem interrompê-lo?	Gli altri la lasciano parlare senza interromperla?
	T2: Gli altri ti lasciano parlare senza interromperti?			
	T3: Gli altri la lasciano parlare, senza interromperla?			
7. Você aproveita as oportunidades de comunicação?	T1: Approfitta delle opportunità comunicative?	Usufruisce/Sfrutta le opportunità di comunicazione che le si presentano?	R: Você utiliza / aproveita as oportunidades de comunicação que se apresentam?	Approfitta delle opportunità di comunicazione che le si presentano?
	T2: Usufruisci delle opportunità di comunicazione?			
	T3: Sfrutta le opportunità di comunicazione che le si presentano?			
8. Os outros aceitam suas sugestões, críticas ou *feedback* (opinião sua sobre os outros)?	T1: Gli altri accettano i suoi consigli, le sue critiche o i suoi feedback (sue opinioni sugli altri)	Gli altri accettano i suoi suggerimenti, le sue critiche o i suoi feedback/riscontri (ovvero la sua opinione sugli altri)?	R: Os outros aceitam suas sugestões, suas críticas ou seus feedback (ou seja, a sua opinião sobre os outros)?	Gli altri accettano i suoi suggerimenti, le sue critiche o i suoi feedback (ovvero la sua opinione sugli altri)?
	T2: Gli altri accettano i tuoi suggerimenti, critiche o feedback (la tua opinione sugli altri)?			
	T3: Gli altri accettano i suoi suggerimenti, le sue critiche o i suoi feedback (ovvero la sua opinione sugli altri)?			
9. Você usa a comunicação como parte do seu *marketing* pessoal?	T1: Utilizza la sua voce come parte del suo marketing personale?	Usa la comunicazione come parte del suo marketing personale?	R: Você usa a comunicação como parte do seu marketing pessoal?	Usa la comunicazione come parte del suo marketing personale?
	T2: Usi la comunicazione come parte del tuo marketing personale?			
	T3: Usa la comunicazione come parte del suo marketing personale?			
10. Você deixa o outro falar sem interrompê-lo?	T1: Lascia che gli altri parlino senza interromperli?	Lascia parlare gli altri senza interromperli?	R: Você deixa os outros falarem sem interrompê-los?	Lascia parlare gli altri senza interromperli?
	T2: Lasci parlare qualcun altro senza interromperlo?			
	T3: Lascia parlare gli altri senza interromperli?			
11. Você presta atenção na mensagem verbal e não verbal do que é dito (voz, linguagem corporal e gestos)?	T1: Presta attenzione al messaggio verbale e non verbale di quello che viene detto (voce, linguaggio corporeo e gestualità)?	Presta attenzione al messaggio verbale e non verbale di quello che viene detto (voce, linguaggio del corpo e gesti)?	R: Você presta atenção na mensagem verbal e não verbal do que está sendo dito (voz, linguagem do corpo e gestos)?	Presta attenzione al messaggio verbale e non verbale di quello che viene detto (voce, linguaggio del corpo e gestualità)?
	T2: Presti attenzione al messaggio verbale e non verbale di ciò che viene detto (voce, linguaggio del corpo e gesti)?			
	T3: Presta attenzione ai messaggi verbali e non verbali di ciò che viene detto (voce, linguaggio del corpo e gesti)?			
12. Você assume o que diz?	T1: Crede in ciò che dice?	Lei si prende la responsabilità di quello che dice?	R: Você assume a responsabilidade daquilo que diz?	Si prende la responsabilità di ciò che dice?
	T2: Prendi la responsabilità per quello che dici?			
	T3: Lei si riconosce in ciò che dice?			
13. Você focaliza a atenção na pessoa interlocutora (evitando ouvir conversas paralelas)?	T1: Focalizza l’attenzione sul suo interlocutore (evitando di ascoltare conversazioni parallele)?	Focalizza l’attenzione sull’interlocutore (evitando di ascoltare conversazioni parallele)?	R: Você focaliza a atenção no interlocutor (evitando de ouvir conversas paralelas)?	Focalizza l’attenzione sull’interlocutore (evitando di ascoltare conversazioni parallele)?
	T2: Focalizzi l'attenzione sull'interlocutore (evitando di ascoltare conversazioni secondarie)?			
	T3: Focalizza l’attenzione sull’interlocutore (evitando di ascoltare conversazioni parallele)?			
14. Você mantém a atenção no discurso da outra pessoa (evitando distrair-se com seus próprios pensamentos)?	T1: Riesce a mantenere l’attenzione su ciò che le dice l’altra persona (evitando che i suoi pensieri la distraggano)?	Mantiene l’attenzione sul discorso dell’altra persona (evitando di distrarsi con i propri pensieri)?	R: Você mantém a atenção no discurso da outra pessoa (evitando de se distrair com seus próprios pensamentos)?	Mantiene l’attenzione su ciò che le dicono (evitando di distrarsi con i propri pensieri)?
	T2: Concentri l’attenzione sul discorso dell'altra persona (evitando di distrarti con i tuoi propri pensieri)?			
	T3: Mantiene l’attenzione sul discorso dell’altra persona (evitando di distrarsi con i propri pensieri)?			
15. Você responde diretamente ao que é perguntado (sem rodeios)?	T1: Risponde direttamente a ciò che le viene chiesto (senza giri di parole)?	Risponde direttamente a ciò che le viene chiesto (senza giri di parole)?	R: Você responde diretamente ao que te vem perguntado (sem meias palavras)?	Risponde direttamente a ciò che le viene chiesto (senza giri di parole)?
	T2: Rispondi direttamente a quello che ti vieni chiesto (senza mezzi termini)?			
	T3: Risponde direttamente a ciò che le viene chiesto (senza giri di parole)?			
16. Você mostra interesse no que está sendo dito, por meio do olhar, postura ou sinais de apoio e aprovação?	T1: Dimostra interesse per ciò che sta per essere detto, attraverso lo sguardo, la postura o segnali di sostegno e approvazione?	Mostra interesse per ciò che viene detto, tramite lo sguardo, la postura o segnali di supporto ed approvazione?	R: Você mostra interesse pelo que está sendo dito, por meio do olhar, da postura ou de sinais de suporte e aprovação?	Mostra interesse per ciò che viene detto, tramite lo sguardo, la postura o segnali di supporto ed approvazione?
	T2: Mostri interesse per ciò che viene detto, attraverso lo sguardo, la postura o segni di sostegno e di approvazione?			
	T3: Mostra interesse in ciò che viene detto, tramite lo sguardo, la postura o segnali di supporto ed approvazione?			
17. Você repete os pontos importantes do que foi dito para se certificar que compreendeu bem?	T1: Ripete i punti chiavi di ciò che è stato detto per essere certo di aver capito bene?	Ripete i punti importanti di ciò che è stato detto per essere sicuro di aver capito bene?	R: Você repete os pontos importantes do que foi dito para estar seguro de ter entendido bem?	Ripete i punti importanti di ciò che le dicono per essere sicuro di aver capito bene?
	T2: Ripeti i punti importanti di ciò che è stato detto per essere sicuri di aver capito bene?			
	T3: Ripete ed analizza i punti più importanti di ciò che le è stato detto per assicurarsi di aver compreso bene?			
18. Você procura memorizar fatos importantes e características da pessoa interlocutora?	T1: Cerca di memorizzare fatti importanti e caratteristiche salienti del suo interlocutore?	Cerca di memorizzare fatti importanti e caratteristiche del suo interlocutore?	R: Você tenta memorizar fatos importantes e características de seu interlocutor?	Cerca di memorizzare fatti importanti e caratteristiche del suo interlocutore?
	T2: Cerchi di memorizzare fatti importanti e caratteristiche dell'interlocutore?			
	T3: Cerca di memorizzare eventi importati e caratteristiche del suo interlocutore?			
19. Você recebe bem críticas, sugestões ou *feedback* (opinião dos outros sobre você)?	T1: Riceve critiche, suggerimenti, feedback (opinione degli altri su di voi)?	Accoglie bene le critiche, i suggerimenti o i feedback (ovvero le opinioni degli altri su di lei)?	R: Você aceita bem as críticas, as sugestões ou os feedback (ou seja, as opiniões dos outros sobre você)?	Accetta le critiche, i suggerimenti o i feedback (ovvero le opinioni degli altri su di lei)?
	T2: Accetti bene critiche, suggerimenti o feedback (l'opinione degli altri su di te)?			
	T3: Accoglie bene le critiche, i suggerimenti o i feedback (ovvero le opinioni degli altri su di lei)?			

**Legenda:** PB= português brasileiro, IT= italiano, T1= tradutor 1 português-italiano; T2= tradutor 2 português-italiano; T3= tradutor 3 português-italiano, R= retrotradução do italiano para o PB, VCI= versão em italiano consenso

A amostra das quatro primeiras etapas foi composta por 8 fonoaudiólogos, sendo seis bilíngues e dois não bilíngues, especialistas no construto. Dentre eles, quatro integravam a equipe de autores do estudo e participaram das etapas 2 e 4. A amostra da quinta etapa foi composta por 101 indivíduos italianos, nascidos e residentes na Itália, que usam a voz e a comunicação oral para desempenharem sua profissão. Para classificar os profissionais da voz, foi utilizada a informação sobre a profissão, autorrelatada pelos participantes do estudo, no formulário de coleta de dados. Utilizou-se a recomendação do COSMIN^(10)^ de tamanho amostral mínimo de 30 indivíduos para o pré-teste.

Os critérios de elegibilidade dos quatro fonoaudiólogos responsáveis pelas etapas de tradução e retrotradução foram: ser fonoaudiólogo bilíngue Italiano-PB, com competências linguísticas nas duas línguas, e conhecimento sobre o construto e a cultura de ambos os países (Itália e Brasil). Os participantes foram selecionados por conveniência e mediante análise do currículo, avaliando a experiência profissional na área da voz e a vivência em ambos os países, por meio de atividades relacionadas à prática da fonoaudiologia, além de entrevista telefônica realizada em ambas as línguas. Para a quinta etapa, a de pré-teste, profissionais italianos de várias áreas, que empregam a voz e a comunicação oral como principal instrumento de trabalho, com idade a partir de 18 anos, atualmente residentes na Itália, foram recrutados por meio de um convite divulgado nas redes sociais. Foram excluídas pessoas em situação de desemprego e/ou que não atuavam em áreas cuja voz desempenha um papel central. Ao clicar no *link* constante no convite, o participante acessava diretamente o questionário de elegibilidade, disponibilizado no Google Drive, ao fim do qual constava a assinatura do Termo de Consentimento Livre e Esclarecido – TCLE. Dos 198 participantes voluntários, 101 foram selecionados após o cumprimento dos critérios de elegibilidade.

Na primeira etapa, o TACCom foi traduzido para o italiano por três fonoaudiólogos. A tradução do título, das instruções, dos itens e das opções da chave de resposta foi realizada respeitando o formato, o conteúdo e os conceitos da versão original, embora com adaptações de aspectos culturais e linguísticos concernentes ao idioma italiano. Essa etapa foi independente e blindada, sem alguma interação entre os participantes.

Na segunda etapa, a de síntese, as três versões traduzidas foram comparadas e sobrepostas por três dos autores deste artigo, decidindo-se sobre a melhor tradução, por consenso, considerando-se as equivalências culturais e semânticas. A primeira versão em italiano do TACCom foi assim construída, e validada por todos os autores do presente artigo.

A terceira etapa consistiu em enviar a versão consenso em italiano para um quarto fonoaudiólogo, que não participou das etapas anteriores, e que não teve acesso à versão original do instrumento, a fim de que realizasse a retrotradução para o PB, sua língua original.

Na quarta etapa, foi constituído o comitê de especialistas, formado por quatro fonoaudiólogos, sendo um especialista em metodologia e três especialistas em voz, com pelo menos cinco anos desde a conclusão da graduação, autores e não autores do presente estudo. O comitê realizou a comparação da versão retrotraduzida com o instrumento original, da qual emergiram apenas divergências lexicais, e, no título, também uma divergência conceitual. Após análise, decidiu-se, por consenso, sobre as mudanças necessárias para a versão final, com equivalências semântica, conceitual, idiomática, experiencial, cultural e operacional. A versão em italiano do TACCom recebeu o nome de *Test di Autovalutazione della Competenza nella Comunicazione* (TACCom-IT). A Figura 1 e o Quadro 1 ilustram as quatro fases do processo de validação transcultural do TACCom para o TACCom-IT.

**Figura 1:** Fluxograma do processo de validação transcultural do TACCom para o italiano (TACCom-IT) segundo as recomendações do COSMIN

**Legenda:** PB= português brasileiro, T1= tradutor 1 português-italiano; T2= tradutor 2 português-italiano; T3= tradutor 3 português-italiano; VCI= versão em italiano consenso; R= retrotradução do italiano para o PB

Na etapa do pré-teste, 101 indivíduos italianos responderam ao TACCom-IT. A média de idade dos participantes foi de 40 anos, sendo 22 homens e 79 mulheres, de várias áreas que utilizam a voz e a comunicação oral como principal instrumento de trabalho. As categorias de profissionais foram: empresários, advogados, gestores, representantes comerciais, professores e profissionais da saúde.

Nessa fase, todos os participantes responderam à versão TACCom-IT. O TACCom^(7)^ original é um instrumento de autoavaliação da competência na comunicação, com 19 itens e chaves de resposta dicotômica (sim/não). Os itens dizem respeito às habilidades de fala e escuta na comunicação interpessoal. O cálculo estatístico das respostas é realizado através de uma planilha específica, dado que a validação ocorreu por Teoria de Resposta ao Item^(9)^. Para essa fase pré-teste, na chave de resposta dicotômica (sim/não) foi adicionada a opção <não aplicável>, traduzida como <*la domanda non è chiara*>. Essa opção deveria ser assinalada caso os participantes não compreendessem o item, ou este não se aplicasse à cultura italiana. Além disso, foi adicionado um espaço para comentários, no caso de o participante responder “não aplicável”, embora não fosse obrigatório preenchê-lo. O número de participantes que respondeu às opções da chave de resposta dicotômica foi comparado ao número daqueles que responderam à opção “não aplicável”, por meio do Teste de Igualdade de Duas Proporções. A análise foi realizada com o IBM SPSS Statistics 29.0.

## RESULTADOS

Na segunda etapa, a da síntese das três versões traduzidas em italiano em uma primeira versão, foi possível obter consenso, em relação às escolhas lexicais, entre pelo menos dois fonoaudiólogos para o título, as instruções, as opções de chave de resposta e em 13 itens. Nos itens 2, 4, 7, 12, 17 e 19 não houve consenso a nível lexical; portanto, optou-se por uma das três traduções.

Na retrotradução, não houve concordância conceitual em relação ao título e às instruções, mas houve concordância quanto às opções das chaves de resposta, nos itens 8, 9, 10, 13 e 14. Nos itens 1, 2, 3, 4, 5, 6, 11, 15, 16, 17, 18, 19, não houve concordância lexical. Em dois itens, 7 e 12, o quarto fonoaudiólogo adicionou uma frase, respectivamente, “que se apresentam” e “a responsabilidade daquilo que diz”.

Na quarta etapa, o comitê de especialistas decidiu adicionar ao título a sigla IT (para referir-se à versão italiana do TACCom); além disso, foi necessário modificar uma expressão no título da versão italiana, de *Test di Autovalutazione della Competenza Comunicativa* para *Test di Autovalutazione della Competenza nella Comunicazione* ***,*** a fim de atender aos critérios de equivalência semântica. Manteve-se a tradução das instruções de acordo com a versão de síntese. Nos itens 2, 7, e 19 foi corrigida uma palavra por item para atender aos critérios de equivalência cultural. Nos demais itens obteve-se equivalências cultural e linguística satisfatórias.

Após a conclusão da etapa com base nas modificações e ajustes do comitê de especialistas, uma segunda versão transculturalmente validada do TACCom-IT foi construída. Todos os dados descritos ao longo das quatro etapas iniciais podem ser observados no Quadro 1.

Na etapa de pré-teste (Tabela 1), não foi possível analisar estatisticamente os itens 3, 10 e 12, visto que todos os participantes responderam com uma opção da chave de respostas habitual do instrumento (sim/não). Para todos os demais itens houve proporção significativamente maior de participantes que responderam com opções da chave de resposta habitual do instrumento (sim/não), comparados à proporção de participantes que responderam <não aplicável> (p<0,001 para todos).

**Tabela 1:** Comparação da proporção de participantes que responderam com as chaves de resposta habituais e com a chave de resposta não aplicável

Item	Sim/ Não		Não aplicável		p-valor
	n	%	n	%	
1. Riesce a catturare e mantenere l’attenzione di chi la ascolta?	100	99,01	1	0,99	<0,001*
2. La sua voce è buona ed espressiva?	97	96,04	4	3,96	<0,001*
3. Parla in modo chiaro, con una buona dizione?	101	100	0	0	
4. Considera facile influenzare gli altri attraverso il suo modo di comunicare?	99	98,02	2	1,98	<0,001*
5. Le persone si ricordano di ciò che ha detto?	95	94,06	6	5,94	<0,001*
6. Gli altri la lasciano parlare senza interromperla?	98	97,03	3	2,97	<0,001*
7. Approfitta delle opportunità di comunicazione che le si presentano?	92	91,09	9	8,91	<0,001*
8. Gli altri accettano i suoi suggerimenti, le sue critiche o i suoi feedback (ovvero la sua opinione sugli altri)?	92	91,09	9	8,91	<0,001*
9. Usa la comunicazione come parte del suo marketing personale?	93	92,08	8	7,92	<0,001*
10. Lascia parlare gli altri senza interromperli?	101	100	0	0	
11. Presta attenzione al messaggio verbale e non verbale di quello che viene detto (voce, linguaggio del corpo e gestualità)?	100	99,01	1	0,99	<0,001*
12. Si prende la responsabilità di ciò che dice?	101	100	0	0	
13. Focalizza l’attenzione sull’interlocutore (evitando di ascoltare conversazioni parallele)?	98	97,03	3	2,97	<0,001*
14. Mantiene l’attenzione su ciò che le dicono (evitando di distrarsi con i propri pensieri)?	98	97,03	3	2,97	<0,001*
15. Risponde direttamente a ciò che le viene chiesto (senza giri di parole)?	98	97,03	3	2,97	<0,001*
16. Mostra interesse per ciò che viene detto, tramite lo sguardo, la postura o segnali di supporto ed approvazione?	100	99,01	1	0,99	<0,001*
17. Ripete i punti importanti di ciò che le dicono per essere sicuro di aver capito bene?	99	98,02	2	1,98	<0,001*
18. Cerca di memorizzare fatti importanti e caratteristiche del suo interlocutore?	100	99,01	1	0,99	<0,001*
19. Accetta le critiche, i suggerimenti o i feedback (ovvero le opinioni degli altri su di lei)?	100	99,01	1	0,99	<0,001^*^

* p-valores obtidos pelo Teste de Igualdade de Duas Proporções

**Legenda:** n=frequência absoluta; %=frequência relativa percentual

Com relação à interpretabilidade, observou-se que, durante a aplicação do pré-teste, alguns participantes selecionaram a opção <não aplicável>, ou seja <*la domanda non è chiara*>, justificando, no espaço disponível para comentários, que a escolha não se referia à clareza ou adequação cultural do item, mas à dificuldade de assinalar uma resposta dicotômica, um ‘não’ ou ‘sim’ absolutos, porque alguns comportamentos de comunicação podem variar de acordo com o interlocutor e o contexto. O comitê de especialistas, após a análise desses comentários, decidiu, na etapa de interpretabilidade, manter as instruções da versão consenso, adicionando, porém, uma frase que parafraseia as instruções, garantindo a equivalência cultural. Assim, a versão final do TACCom-IT foi consolidada (Apêndice A).

## DISCUSSÃO

No ambiente dinâmico e competitivo do mercado de trabalho atual, diversas *soft skills*, ou habilidades socioemocionais contribuem para o sucesso das organizações. Dentre elas, está a habilidade de competência na comunicação. Sua importância é tão grande, que alguns autores sugerem o emprego da expressão *essential skill* ao invés de *soft skill* para se referir a essa competência, muito requisitada nos altos cargos das organizações, e cuja ausência pode influenciar negativamente a chance de uma promoção ou até mesmo contribuir para a demissão de um profissional^(8)^.

A competência na comunicação, no mundo das organizações, nem sempre é uma exigência na fase inicial de desenvolvimento de carreira, porém, torna-se um recurso imprescindível e um pré-requisito na passagem de uma função operacional para cargos de gestão. Nesse momento, podem ser uma exigência as habilidades conceituais de comunicação, como por exemplo, a capacidade cognitiva de visualizar, entender e coordenar todas as atividades e interesses das organizações em um sistema, compreendendo suas inter-relações^(11,12)^.

Um profissional que se comunique de maneira ponderada, planejada, estruturada, preparada e assertiva tem a capacidade de gerar considerável impacto psicológico no seu ouvinte, causando-lhe a impressão de profissionalismo e segurança, o que espelha clareza de raciocínio e pensamento, além do domínio das regras do mundo das organizações. Por isso, muito se vem buscando pelo desenvolvimento dessa competência em cargos de liderança.

Para que a comunicação seja desenvolvida de forma assertiva e consciente, faz-se necessário dispor de instrumentos avaliativos, que ajudem os profissionais a compreender quais habilidades já existem, quais precisam ser melhoradas e, ainda, quais precisam ser adquiridas. Na língua italiana não há nenhum questionário para análise da percepção da competência na comunicação, o que justifica a validação transcultural para o italiano do TACCom, um instrumento de autoavaliação da competência na comunicação validado em português brasileiro.

Na presente validação transcultural, foram encontradas algumas discordâncias lexicais e conceituais, durante o processo de tradução e retrotradução do TACCom-IT. Esses dados corroboram a importância em seguir etapas bem estruturadas no processo de validação transcultural de um instrumento, e confirmam que uma simples tradução literal não é, de forma alguma, suficiente, pois ignora e desconsidera a complexidade cultural^(13)^.

A equivalência semântica e conceitual indicou a necessidade de ajustes no título e nas instruções, garantindo o mesmo peso semântico da versão original. Em relação ao título, a discussão apurou a melhor expressão, em italiano, para traduzir a frase “competência na comunicação”. Esse conceito pode ser traduzido de diversas formas: *capacità comunicativa, capacità di comunicazione, competenza comunicativa, abilità comunicative, competenza nella comunicazione*. Dentre elas, na fase de síntese do estudo, optou-se por *competenza comunicativa* (competência comunicativa). Todas as opções referem-se a ambos os conceitos: competência linguística ou gramatical e competência de fala e escuta na comunicação. Todavia, em português, a competência comunicativa refere-se exclusivamente à competência linguística ou gramatical para produzir frases que sejam entendidas como produtos não apenas de uma determinada língua, como também coerentes à intenção do falante, em dada circunstância. Envolve também a competência textual, vista como a capacidade do usuário de, em situações de interação comunicativa, produzir, compreender, transformar e classificar textos que se mostrem adequados à interação comunicativa pretendida, seguindo regularidades e princípios de organização e construção dos textos e do funcionamento textual, uma vez que os textos são a unidade da língua em uso. Na fase de análise do comitê de especialistas, por consenso, optou-se por modificar a expressão para *competenza nella comunicazione*, a fim de garantir a equivalência semântica e conceitual *entre o TACCom-IT e o TACCom.*

Nessa mesma fase, para os itens 2, 4, 7, 12, 17 e 19 foram realizados ajustes de palavras, de modo que o significado refletisse aquele do TACCom, e que o sinônimo respeitasse culturalmente o contexto italiano^(14)^.

Na fase pré-teste do TACCom-IT, houve proporção significativamente maior de participantes que responderam com opções da chave de resposta habitual do instrumento, quando comparada à opção <não-aplicável>. Os itens em que não houve diferença foram os que todos os participantes responderam com as opções habituais da chave de resposta do instrumento. Dessa forma, não houve necessidade de modificação e/ou eliminação de itens.

No tocante à interpretabilidade, alguns ajustes foram necessários, levando em consideração os comentários deixados pelos participantes na fase pré-teste. A interpretabilidade refere-se ao grau em que se pode atribuir significado qualitativo para os escores quantitativos de um instrumento ou mudança nos escores quantitativos^(10)^. Observou-se uma característica diferente na população italiana, em que os participantes tiveram dificuldade para escolher entre as opções da chave de resposta original, ‘sim’ e ‘não’. Para resolver esse desconforto, optou-se por manter a chave de resposta original e as instruções da versão consenso, além de acrescentar uma frase, parafraseando as instruções do TACCom-IT. A instrução inicial “*Le seguenti domande si riferiscono a diversi aspetti del parlato e dell’ascolto. Legga attentamente ciascuna domanda e scriva se ha o meno questi comportamenti comunicativi*” foi ajustada para *“Le seguenti domande si riferiscono a diversi aspetti del parlato e dell’ascolto. Legga attentamente ciascuna domanda e scriva se ha o meno questi comportamenti comunicativi. Risponda SI, se questi comportamenti si compiono/sono presenti la maggior parte delle volte e risponda NO, se questi comportamenti non sono presenti la maggior parte delle volte”.* O ajuste foi realizado a fim de garantir a boa interpretabilidade do TACCom-IT e de guiar os participantes na escolha da chave de reposta que mais refletisse o real comportamento comunicativo^(15)^.

Apesar dos resultados satisfatórios na validação transcultural, para garantir a validade, confiabilidade e responsividade do TACCom-IT, será necessário demonstrar as demais propriedades psicométricas do instrumento na população italiana, o que deverá ser feito em estudos futuros.

Como limitação, ressalta-se que a descrição sociodemográfica da amostra foi restrita às variáveis idade, sexo e profissão. A inclusão de informações adicionais poderia enriquecer a caracterização do grupo investigado.

## CONCLUSÃO

A versão validada transculturalmente para o italiano do TACCom foi denominada *Test di Autovalutazione della Competenza nella Comunicazione* (TACCom-IT) e manteve a estrutura original do instrumento. Contudo, além dos ajustes nos processos habituais de validação transcultural, foi preciso uma adaptação das instruções do teste, para que se pudessem obter todas as equivalências necessárias. O estudo apresentou resultados satisfatórios em relação à validação transcultural do TACCom-IT.

## Apêndice A Versão final, transculturalmente validada do TACCom

TEST DI AUTOVALUTAZIONE DELLA COMPETENZA NELLA COMUNICAZIONE (TACCom-IT)

Nome: ______________________________________ Data: ___/___/____

Le seguenti domande si riferiscono a diversi aspetti del parlato e dell’ascolto. Legga attentamente ciascuna domanda e scriva se ha o meno questi comportamenti comunicativi. Risponda SI, se questi comportamenti si compiono/sono presenti la maggior parte delle volte e risponda NO, se questi comportamenti non sono presenti la maggior parte delle volte

**Table:**

1. Riesce a catturare e mantenere l’attenzione di chi la ascolta?	sì	no
2. La sua voce è buona ed espressiva?	sì	no
3. Parla in modo chiaro, con una buona dizione?	sì	no
4. Considera facile influenzare gli altri attraverso il suo modo di comunicare?	sì	no
5. Le persone si ricordano di ciò che ha detto?	sì	no
6. Gli altri la lasciano parlare senza interromperla?	sì	no
7. Approfitta delle opportunità di comunicazione che le si presentano?	sì	no
8. Gli altri accettano i suoi suggerimenti, le sue critiche o i suoi *feedback* (ovvero la sua opinione sugli altri)?	sì	no
9. Usa la comunicazione come parte del suo *marketing* personale?	sì	no
10. Lascia parlare gli altri senza interromperli?	sì	no
11. Presta attenzione al messaggio verbale e non verbale di quello che viene detto (voce, linguaggio del corpo e gestualità)?	sì	no
12. Si prende la responsabilità di ciò che dice?	sì	no
13. Focalizza l’attenzione sull’interlocutore (evitando di ascoltare conversazioni parallele)?	sì	no
14. Mantiene l’attenzione su ciò che le dicono (evitando di distrarsi con i propri pensieri)?	sì	no
15. Risponde direttamente a ciò che le viene chiesto (senza giri di parole)?	sì	no
16. Mostra interesse per ciò che viene detto, tramite lo sguardo, la postura o segnali di supporto ed approvazione?	sì	no
17. Ripete i punti importanti di ciò che le dicono per essere sicuro di aver capito bene?	sì	no
18. Cerca di memorizzare fatti importanti e caratteristiche del suo interlocutore?	sì	no
19. Accetta le critiche, i suggerimenti o i *feedback* (ovvero le opinioni degli altri su di lei)?	sì	no
